# Exploring the Power of Thermosonication: A Comprehensive Review of Its Applications and Impact in the Food Industry

**DOI:** 10.3390/foods12071459

**Published:** 2023-03-29

**Authors:** Alaa R. Abdulstar, Ammar B. Altemimi, Asaad R. Al-Hilphy

**Affiliations:** Department of Food Science, College of Agriculture, University of Basrah, Basrah 61004, Iraq

**Keywords:** TS, food processing, quality parameters, novel technologies, microbial inactivation

## Abstract

Thermosonication (TS) has been identified as a smart remedy for the shortcomings of heat treatment, which typically requires prolonged exposure to high temperatures. This technique combines moderate heat treatment with acoustic energy to eliminate harmful microorganisms and enzymes in food products. Unlike conventional heat treatment, thermosonication utilizes short holding times, allowing for the preservation of food products’ phytochemical compounds and sensory characteristics. The benefits and challenges of this emerging technology, such as equipment cost, limited availability of data, inconsistent results, high energy consumption, and scale-up challenges, have been assessed, and the design process for using ultrasound in combination with mild thermal treatment has been discussed. TS has proven to be a promising technique for eliminating microorganisms and enzymes without compromising the nutritional or sensory quality of food products. Utilizing natural antimicrobial agents such as ascorbic acid, Nisin, and ε-polylysine (ε-PL) in combination with thermosonication is a promising approach to enhancing the safety and shelf life of food products. Further research is required to enhance the utilization of natural antimicrobial agents and to acquire a more comprehensive comprehension of their impact on the safety and quality of food products.

## 1. Introduction

Foods are composed of numerous constituents, including proteins, vitamins, carbohydrates, fats, minerals, water, and other organic substances, each with their individual compositions [1]. Previous research indicates that the traditional technique of pasteurization, which is referred to as conventional pasteurization (CP), has been utilized for a significant period to safeguard the safety and extend the shelf life of various kinds of foods and drinks by eliminating microorganisms [2,3,4]. However, Numerous published papers demonstrated that CP possesses adverse impacts on the flavor, consistency, aroma, appearance, and nutritional quality of processed foods. [5,6]. In response to the increasing desire among consumers for good products, novel methods of preserving and processing food have been created and experimented with. One of these innovative techniques is Ultrasound, which may serve as an alternative way to process and conserve food [7].

Previous research has pointed out that ultrasound waves are formed by converting electrical energy to mechanical energy by utilizing piezoelectric materials [8]. While liquid foods are being processed, ultrasound waves propagate through the liquid, causing alterations in pressure that result in the creation of bubbles. As a result of subsequent compression cycles, these bubbles collapse with great force, generating zones of high temperature and pressure that are known as cavitation [9]. When heat is applied to the ultrasonic waves, with temperatures exceeding 50 °C, it is referred to as TS [10].

TS is a method that uses a combination of ultrasound and heat to moderately heat a product. Studies have demonstrated that TS is a successful technique for deactivating various types of microorganisms., including bacteria, viruses, yeasts, and molds. According to [11], adding heat to sonication (thermosonication) is more effective in killing microbes compared to using sonication alone [12].

The method has also been employed to cure a range of food items, such as fruit beverages [12], milk and human milk [13,14], soy milk [15], beer [16], and wine [17]. Studies have shown that TS is a highly effective substitute for conventional thermal processing techniques, as it can quickly decrease the microbial load of food products by five logarithmic units in just seconds [14].

TS has the capability to retain the sensory and nutritional characteristics of food products, which sets it apart from conventional thermal processing techniques as a major advantage [18,19]. In contrast, TS has been demonstrated to have little impact on the taste and nutritional characteristics of food items, making it an attractive option for manufacturers who want to produce high-quality, minimally processed foods. Another advantage of TS is its ability to reduce processing time and energy costs. Traditional thermal processing methods can be time-consuming and energy-intensive, requiring prolonged heating time and high temperatures.

Therefore, the main aim of this paper was to provide a comprehensive review of the applications and impact of TS in the food industry; this topic can bring new insights and knowledge to the readers. It can help understand the potential benefits and limitations of this technology and its impact on the food industry. Moreover, the topic can highlight the current research trends and future prospects of TS, which can inspire new research ideas and innovations. Overall, exploring the power of TS can contribute to the advancement of the food industry by providing an alternative approach to conventional food processing technologies.

## 2. Ultrasound Generation

Ultrasound is a term used to describe sound waves that have a frequency higher than 20 kHz, a level that exceeds the range of human hearing [20]. There are multiple methods to produce ultrasound, including piezoelectric transducers, magnetostrictive transducers, and electrostatic transducers [20]. Ultrasound waves are generated using a device called an ultrasound transducer, which converts electrical energy into sound waves. The transducer contains a piezoelectric crystal, which vibrates in response to an electric current. As the crystal vibrates, it creates sound waves that propagate through a medium, such as air or water [21]. The most prevalent type of transducer used in ultrasound imaging is the piezoelectric one. These transducers employ a piezoelectric crystal that receives an alternating electric field, causing it to vibrate and generate ultrasound waves. The opposite is also true; when ultrasound waves are applied to a piezoelectric crystal, it generates an electric field that can be measured [22].

The fluid-driven transducer operates by propelling liquid through a thin metal blade to create high-frequency vibrations suitable for mixing and homogenization applications. The magnetostrictive transducer uses ferromagnetic materials that change their size when exposed to a magnetic field. This alteration creates the mechanical vibrations needed for the transducer to function. However, The efficiency of this system is comparatively low, with 60% of the energy transferred to acoustic energy [23]. Piezoelectric transducers, on the other hand, by responding to electrical signals, modify the dimensions of piezoceramic materials such as lead zirconate titanate, barium titanate, and lead metaniobate to generate sound energy. The most commonly employed devices exhibit higher efficiency rates, converting a significant portion of the energy (ranging from 80% to 95%) into acoustic energy [23,24].

To convey ultrasonic vibrations to a specimen, a coupler device is employed in an application system. This device can be an ultrasonic bath or a probe system. In ultrasonic baths, the transducers are commonly attached to the bottom of the container, and most of them function at around 40 kHz. In contrast, probe systems use horns or probes to deliver and intensify the ultrasonic signal. The length of the horns or probes needs to be half the wavelength or a multiple of it to keep the resonant conditions within the system. The shape of the horn decides the amplification of the ultrasonic signal. If the probe has a uniform diameter across its length, no amplification will take place, but the acoustic energy will still get transmitted to the medium [23,24,25].

## 3. Classification of Ultrasonication Application

Currently, the utilization of ultrasound is becoming more and more prevalent in the food sector. Ultrasound devices are easily accessible and are deemed sustainable and eco-friendly technology. The application of ultrasound can be performed in three primary ways, which include direct application to the product, coupling with a device, or immersion in an ultrasonic bath [26]. Ultrasound can be classified based on its frequency, intensity, and mode of application. Low-frequency ultrasound (<1 MHz) is used for therapeutic purposes, such as tissue heating and disruption of cell membranes [27]. High-frequency ultrasound (>10 MHz) is used for diagnostic purposes, such as medical imaging [28]. Ultrasound intensity is measured in units of watts per square centimeter (W/cm^2^) and can range from 0.001 W/cm^2^ for diagnostic imaging to 10 W/cm^2^ for therapeutic applications [29]. The mode of application of ultrasound can be continuous or pulsed. Pulsed ultrasound is used for therapeutic purposes, where the ultrasound waves are applied in short bursts with a period of rest in between, while continuous ultrasound is used for diagnostic purposes, where the ultrasound waves are applied continuously [30,31,32].

Ultrasound has numerous applications in various fields, including medicine, industry, and material science. In medicine, ultrasound is used for diagnostic imaging, therapeutic purposes, and drug delivery [33]. Diagnostic ultrasound imaging utilizes high-frequency ultrasound waves to produce pictures of the body’s internal structures, including organs, tissues, and blood vessels [34]. Therapeutic ultrasound uses low-frequency for various therapeutic purposes, such as tissue heating, tissue disruption, and wound healing. Ultrasound can also be used for drug delivery, where Ultrasound waves are employed to improve the transportation of medications to particularly targeted tissues [35].

In the industrial sector, ultrasound is used for various purposes, including cleaning, welding, and cutting [36,37]. Ultrasonic cleaning employs high-frequency to remove dirt, grease, and other contaminants from surfaces. Using ultrasound waves, ultrasonic welding fuses multiple materials together, while ultrasonic cutting severs materials such as plastics and metals with the aid of high-frequency ultrasound waves [38].

## 4. Thermosonication (TS)

TS, also known as ultrasound-assisted thermal processing or sono-thermal processing, is a method of preserving food that involves using both heat and ultrasound to decrease the number of microorganisms present in the food [39]. In recent times, this technique has gained popularity because it can efficiently remove microorganisms from food while maintaining the food’s nutritional and sensory properties [18]. The process of TS involves applying ultrasound waves to a liquid while it is being heated [40]. The ultrasound waves create cavitation bubbles within the food, which cause mechanical stress and lead to the formation of micro-streams and high shear forces [41]. These mechanical impacts interfere with the cell membranes of microorganisms, ultimately causing them to lose their ability to function [42].

Research has demonstrated that TS can successfully decrease the number of various types of microorganisms, such as bacteria, viruses, and fungi [43]. For example, In their research on kiwi peel, Boghossian et al. [44] investigated the impact of TS on Listeria and discovered that the combination of heat and ultrasound had a cooperative impact on eliminating the bacteria, as evidenced by a notable decrease in the D-values.

Preserving the nutritional and sensory characteristics of the treated food is a significant advantage of using TS [45]. Conventional heat treatment techniques, such as pasteurization, may cause the deterioration of vitamins, enzymes, and other nutrients present in the food [46]. However, TS is a viable substitute for thermal processing when it comes to fruit juices, such as blackberry and orange juices, as it has a minimal effect on their quality [47,48]. Refs. [49,50,51] also studied the effects of TS on watermelon juice, tomato juice, and watercress, respectively, to improve their quality.

Research has demonstrated that TS may have uses in the food industry beyond just preservation. For example, The quality of phytochemical extracts and energy efficiency of TS has been evaluated, comparing it with conventional solid–liquid extraction methods such as percolation and Soxhlet [52]. The investigation revealed that TS-assisted extraction techniques could reach high temperatures due to the combination of acoustic cavitation and external heating. TS carried out at 400 W resulted in greater temperature differentials [52]. Nonetheless, the intensity of the acoustic cavitation decreased at 60 °C, which led to lower temperature differentials in TS. The extraction of phenolic and antioxidant compounds was more effective with TS at 300 W and 60 °C, resulting in higher levels of these compounds compared to percolation and Soxhlet extraction methods. In particular, TS for 20 min at 300 W and 60 °C was found to recover more total phenolic compounds than percolation and was only slightly less effective than Soxhlet extraction. Additionally, TS was more energy-efficient due to the reduced extraction time and solvent volume needed. Therefore, TS has the potential to be a valuable technique for recovering and making bioactive compounds available [52].

## 5. Basic Principle of TS

**Figure 1:**
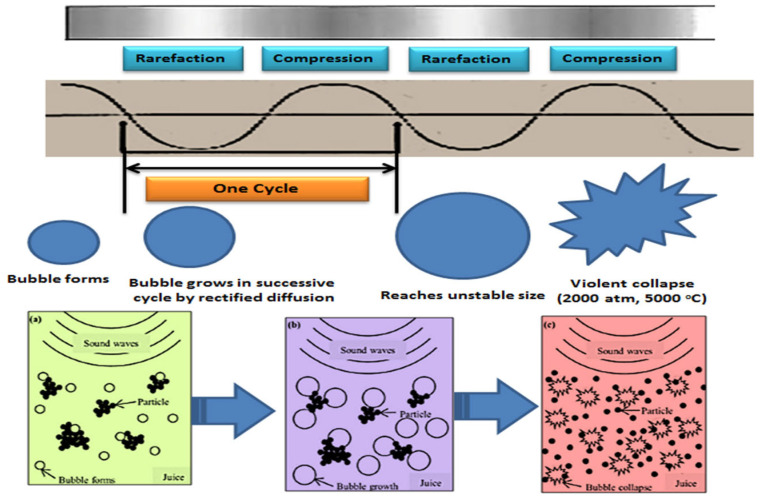
The phenomenon of cavitation. (**a**) the creation of bubbles in juice through the use of sound waves; (**b**) the expansion of bubbles until they reach their maximum size; and (**c**) the collapsing of bubbles, resulting in the dispersion of particles and the occurrence of cell disruption.

The process of cavitation, including the development, expansion, and collapse of gas bubbles, is illustrated in Figure 1. Before cavitation occurs, ultrasonic waves generate gas bubbles within a liquid. The oscillation of these gas bubbles during cavitation leads to modifications in their surroundings, which enable solvent vapor and dissolved gas to enter and leave the bubbles. The expansion of the bubbles during the compression stage of the oscillations results in them taking in more gas and vapor than they discharge, leading to their enlargement to an unstable extent. Eventually, the bubbles burst during compression, creating high pressure and heat that can break down compounds in the liquid and produce a localized sterilization effect. Between ultrasonic waves, thermosonication passes through the liquid and dissolved gas, causing particle dispersion and cell disruption [53].

## 6. Advantages and Disadvantages of TS

This technique has been used in various industries, including food processing, chemical synthesis, and environmental remediation. In this paper, we discussed the advantages and disadvantages of TS as follow:

### 6.1. Advantages

#### 6.1.1. Increased Efficiency

TS can significantly increase the efficiency of various processes. When heat and ultrasound are used together, they can result in cavitation. This phenomenon creates bubbles, and when these bubbles collapse, it produces high temperatures and pressure. This can break down materials into smaller particles and increase the rate of chemical reactions. As a result of this, the processing time is accelerated, and there is an increase in yields [54,55].

#### 6.1.2. Improved Quality

TS can also improve the quality of products. By breaking down materials into smaller particles, it can improve the uniformity of materials, resulting in a more homogeneous product. Additionally, the high temperatures and pressure generated by the technique can also sterilize materials [56,57].

#### 6.1.3. Environmentally Friendly

TS is an environmentally friendly technique as it reduces the need for harsh chemicals and solvents. The combination of heat and ultrasound can break down materials without the need for additional chemicals, reducing waste and lowering the environmental impact of the process [26,58].

#### 6.1.4. Cost-Effective

TS can also be cost-effective compared to traditional processing techniques. The faster processing time and increased yields can lead to reduced production costs. Additionally, the reduced need for additional chemicals and solvents can also lower costs [59,60].

### 6.2. Disadvantages

#### 6.2.1. Equipment Costs

The expense of equipment is a significant drawback of TS. TS requires specialized equipment that can generate high temperatures and pressure, which can be expensive to purchase and maintain. The high cost of equipment can limit the accessibility of the technique for small businesses and laboratories [61].

#### 6.2.2. Temperature Control

TS requires precise temperature control to avoid damaging the materials being processed. The high temperatures generated by the technique can lead to the denaturation of proteins and other heat-sensitive materials. Precise temperature control is necessary to prevent this from happening and ensure that the materials being processed maintain their integrity [62,63]. 

#### 6.2.3. Limited Applicability

TS may not be suitable for all materials and processes. The high temperatures and pressure generated by the technique may not be suitable for some materials, and the cavitation process may not be effective in breaking down certain materials. This limits the applicability of the technique and may require alternative processing techniques to be used.

#### 6.2.4. Safety Concerns

TS can also pose safety concerns. The high temperatures and pressure generated by the technique can be dangerous if not properly controlled. Additionally, the use of ultrasound can also pose a risk to operators if they are not properly trained in its use [60].

## 7. Impact of TS on the Quality of Food Items

The food industry places great importance on food processing, as the quality of processed food products is heavily influenced by the methods used. In recent years, TS has emerged as a popular food processing technique. TS involves the simultaneous application of heat and ultrasound to food products. This technique has been shown to have several benefits, including improved shelf life, enhanced nutrient retention, and improved product quality.

### 7.1. Nutrient Retention

TS has demonstrated the ability to enhance the preservation of vital nutrients present in food products [64]. Research has shown that the application of TS led to a boost in the levels of ascorbic acid by 11.40% to 18.55%, total phenolic content by 17.98% to 18.35%, carotenoids by 2.19% to 4.30%, flavonoids by 10% to 16%, and antioxidant activity by 32.52% to 48.5% [65]. Thermosonicated kutkura juice samples showed minimal changes in nutrient retention compared to both fresh and thermally treated juices. The investigation revealed that thermosonicated juice exhibited higher levels of total phenolic content (476.32–579.87) mg gallic acid equivalents (GAE)/mL), total flavonoid content (71.42–99.89 mg quercetin equivalents (QE)/mL), and antioxidant activity (65.10–83.18%) compared to pasteurized (total phenolic content—399.83 mg GAE/mL, total flavonoid content—51.02 mg QE/mL, AOA—49.91%) and fresh (total phenolic content—421.21 mg GAE/mL, total flavonoid content—61.38 mg QE/mL, AOA—56.40%) kutkura juices. Furthermore, the research found that the retention of ascorbic acid was higher in thermosonicated juices (27.10–36.42 mg/100 mL) in comparison to pasteurized juices (26.14 mg/100 mL) but lower than that of fresh kutkura juice (37.21 mg/100 mL) [66]. Different ultrasound power levels that affect the quality of yogurt drinks made with thermosonicated milk were evaluated. Raw milk samples were heated to 70 °C and then subjected to ultrasound treatments at varying power levels (100, 125, and 150 W). Yogurt drinks were produced using thermosonicated milk or milk processed using conventional heating (10 min at 90 °C). The study found that the TS treatment did not have a significant effect on the composition or color of the samples. However, it did decrease serum separation values and increase apparent viscosity values at higher ultrasound power levels. All yogurt drink samples exhibited non-Newtonian behavior based on rheological measurements. Overall, the study concluded that TS treatment could be effectively used in the production of yogurt drinks and improve its key quality parameters, such as delaying serum separation and increasing apparent viscosity [67].

### 7.2. Texture

The quality of food is significantly influenced by its texture, and research has demonstrated that TS can enhance the texture of food items [68]. In a study conducted by Peña-Gonzalez et al. [69], beef was subjected to ultrasound treatment with a frequency of 40 kHz and intensity of 11 W/cm^2^ for 60 min after being stored for 0, 7, or 14 days. The researchers found that the tenderness of the beef had increased.

### 7.3. Shelf Life

Research has demonstrated that TS can enhance the longevity of food items [70]. According to Desphande et al. [71], the quality of milk can be improved by TS by reducing the amount of thermophilic and total bacteria in it and prolonging its shelf life. By subjecting the milk to a temperature of 72 °C, TS significantly decreased the number of thermophilic bacterial cells and spores in a batch system by up to one log. Combining TS with heat in a continuous system further reduced *G. stearothermophilus* cells by up to 0.5 log. Although the reduction in bacteria was statistically significant, it may not have a significant impact on the overall quality of milk in practical situations. However, applying TS for 11 s at 72 °C together with pasteurization in a continuous system resulted in a higher log reduction of 4.1, compared to 2.8 in the control group at week 0. Throughout its shelf life, the milk that was subjected to TS had a reduced amount of bacteria compared to the control group [71].

### 7.4. Color

This method has the potential to improve the color of meat products, fruits, and vegetables [72]. Nowacka et al. [73] utilized an ultrasonic bath with a frequency of 21 kHz and a power of 300 W to pre-treat beetroot, varying the duration of the pre-treatment from 10 to 15 min. The study revealed that the color values of beetroot powder did not exhibit significant changes when the pre-treatment duration was increased from 10 to 15 min. However, an increase in red and yellow color values was observed with an increase in pre-treatment duration. The research suggests that the most favorable outcome on color values was obtained with a 15 min ultrasonic treatment.

The effects of ultrasonication on pasta color were studied. Raso and Álvarez Lanzarote [74] conducted research where they used ultrasonic technology operating at a power setting of 50 W, a frequency of 40 kHz, and cooked the product for 8 min. According to their findings, ultrasonic cooking methods were better at preserving the yellow color of the final product, indicating a decrease in pigment loss.

Nevertheless, TS may also result in unfavorable color changes in certain food products. A study demonstrated that the color attributes of barberry juice were significantly affected by TS treatment, with the color values (L*, a*, b*, and C*) of the treated juice being lower than the control. This reduction in color values implies a decrease in color intensity [75]. Santhirasegaram et al. [76] also observed that thermal treatments could cause visible alterations in the color of juice samples, as previously noted in studies such as Lee et al. [77] on heat-treated Valencia orange juice, where a range of 3.0 < delta E < 6.0 was recorded. The research findings indicated that the heat treatment led to a lighter color (higher L value) and a more concentrated juice. However, in the case of sonicated juice, an increase in lightness (L) was noted, along with a decrease in redness (+a) and yellowness (+b) values. The outcome was in line with Tiwari et al. [78] research, where they discovered that sonicated orange juice experienced color degradation due to the accumulation of unstable particles, leading to partial precipitation. The natural pigments in mango juice, mainly carotenoids, determine its color. As a result, high temperatures and sonication can cause accelerated carotenoid isomerization, leading to a loss of yellow color (a reduction in +b value). Additionally, the L value increase causes the juice to become more transparent as previously formed colored compounds are destroyed. The decrease in (+a) and (+b) can be attributed to the increased degree of browning [79,80].

### 7.5. Flavor

The taste and scent of food are caused by a complicated blend of different types of compounds, both volatile and non-volatile, which together create flavor. The quality and sensory properties of food depend largely on its flavor, and therefore, it is essential to optimize the flavor of food products [81]. TS has been shown to improve the flavor of food products. Studies have shown that the technique can enhance the aroma and taste of fruits, vegetables, and meat products. For example, Salehi [82] investigated how a new technique that uses ultrasound instead of heat affected the taste, smell, texture, and overall sensory properties of orange juice. The study found that the sensory characteristics of the juice treated with ultrasound were satisfying and similar to that of freshly squeezed juice. The results of a survey on consumer preferences indicate that ultrasound technology holds great promise for use in the food industry.

Bui [83] investigated how low and high frequency ultrasound affected the formation of volatile compounds and the resulting unpleasant tastes in milk and dairy products. The number of volatile compounds produced was found to be influenced by the conditions of the ultrasound process, such as sonication time, temperature, and frequency. Low-frequency ultrasound generated fewer volatile compounds, while high-frequency ultrasound generated more. Additionally, the composition of milk proteins and fat affected the production of these compound. With careful management and fine-tuning of these variables, it could be achievable to decrease the production of unwanted volatile compounds, remove off-flavors, and encourage the application of ultrasound technology in the dairy sector [83].

## 8. Effects of TS on Inactivation Microorganisms in Food

TS is a process that combines high-intensity ultrasound waves with thermal energy to produce a synergistic effect on microorganisms [66]. The application of ultrasound waves that generate both heat and mechanical pressure can cause considerable harm to the microorganism’s structure, resulting in their deactivation. The following are some of the impacts of TS on microorganism deactivation:

### 8.1. Disruption of Cell Membranes 

The ultrasound waves’ mechanical pressure can rupture or make the cell membranes of microorganisms porous, resulting in the release of components from inside the cells. This action can lead to the destruction of the microorganisms beyond repair [11,84].

### 8.2. Denaturation of Proteins

The thermal energy generated by the process can cause the denaturation of proteins in microorganisms, leading to the disruption of their structure and function. This process can result in the deactivation of enzymes and other essential proteins within the microorganisms [7,85].

### 8.3. Generation of Free Radicals

The process of cavitation can promote the release of free radicals as a result of the sonolysis of water molecules. These free radicals can then react with the amino acid residues of enzymes, which are important for the stability, substrate binding, and catalytic function of the enzyme. This can cause changes in enzymatic activity [86]. Guimarães et al. [87] suggest that cavitation has the ability to interact with free radicals that are created along with cellular DNA, and the bactericidal and bacteriostatic effects of cavitation are due to the production of hydrogen peroxide.

### 8.4. Cavitation

Ultrasound waves have the ability to produce little bubbles or pockets within the liquid, which can implode and create areas of high pressure and temperature in the surrounding area [88]. According to Meroni et al. [88], the use of cavitation bubbles to deactivate microorganisms is more effective than traditional heat treatments. This is because the turbulence created by the bubble collapse can damage microorganism cell membranes and release cellular components. Table 1 below summarizes the percent inactivation of different microorganisms under various TS conditions.

## 9. The Impact of TS on the Bioactive Compounds Present in Food

**Figure 2:**
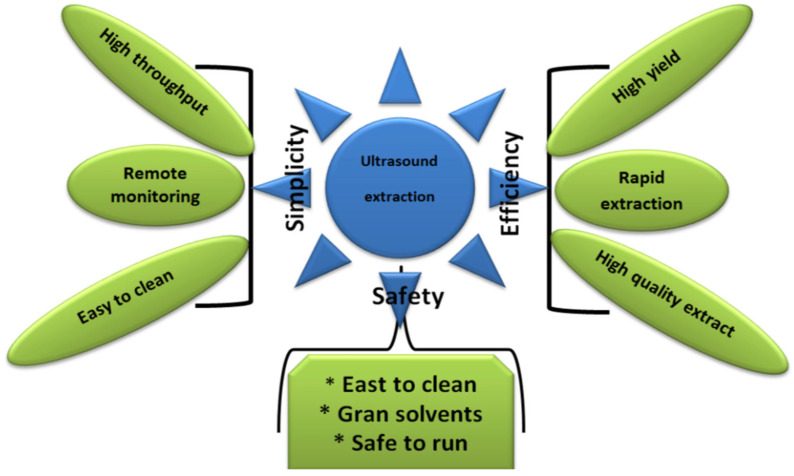
The benefits of using ultrasound technology in order to extract desired compounds.

The utilization of TS technology has been implemented to enhance the extraction and conservation of bioactive compounds in food [97]. Figure 2 illustrates a diagram that explains the reasons why ultrasound extraction is considered a technology with great potential. Bioactive compounds are naturally occurring substances in food that can have positive health effects when consumed [98]. Antioxidants, polyphenols, carotenoids, and flavonoids are some instances of bioactive compounds. The implementation of TS in food processing has demonstrated encouraging outcomes concerning the improvement of the bioavailability and efficiency of these compounds [99].

TS is a technique that utilizes high-frequency sound waves to a food material, either liquid or solid, that is heated at the same time. These sound waves generate pressure waves inside the food material, leading to the extraction of bioactive compounds from the cellular matrix [100]. The heat applied during TS also aids in the release of these compounds by disrupting the cell membranes [101].

The use of TS has demonstrated a remarkable enhancement in the ability to extract bioactive substances from food, thereby improving production rates. Ultrasound technology has been proven to positively impact the characteristics of alcoholic drinks, such as their phytochemical, physicochemical, biological, and sensory properties. One notable effect is the potential to increase anthocyanin content in red wine by as much as 50% while reducing the aging process by more than 90%, thereby increasing production efficiency [102]. The influence of TS on the antioxidant properties of mango juice was also examined.

The results revealed that TS holds considerable potential for increasing the concentration of bioactive substances and antioxidants in mango juice samples, surpassing the effects of ohmic techniques [103]. This increased extraction efficiency can be attributed to the ability of TS to disrupt the cell membranes and release more bioactive compounds into the solvent.

Ultrasonic treatment had a noticeable effect on the physiological properties of cherry juice. Lengthening the treatment duration from 2 to 10 min led to elevated levels of total phenolic content, antioxidant activity, and ascorbic acid in the juice [104]. Xia et al. [105] found that ultrasonic treatment was superior to traditional methods in extracting tea polyphenols, caffeine, theanine, and aromatic compounds. In their research, Qin et al. [106] employed ultrasonic techniques to produce a rice-flavored tea wine using Anhua black tea, which was enriched with probiotics to decrease blood lipid levels. The investigation revealed that ultrasonic parameters such as time, solid/liquid ratio, temperature, and power had an impact on the polyphenol concentration of the black tea wine. A study [107] explored the impact of ultrasonic treatment on the extraction of TPC and radical scavenging activity from black tea prepared using the cold-brew method. The study found that the highest concentration of bioactive compounds was obtained with 69.9% amplitude, 25 mL of solvent volume, and 30 min of sonication time at 4 °C, resulting in a TPC of 78.97 mg GAE/g. In contrast to the conventional brewing method, the ultrasonic brewing process boosted the extraction of phenolic compounds four-fold and shortened the brewing time from 6 h to a mere 30 min.

**Figure 3:**
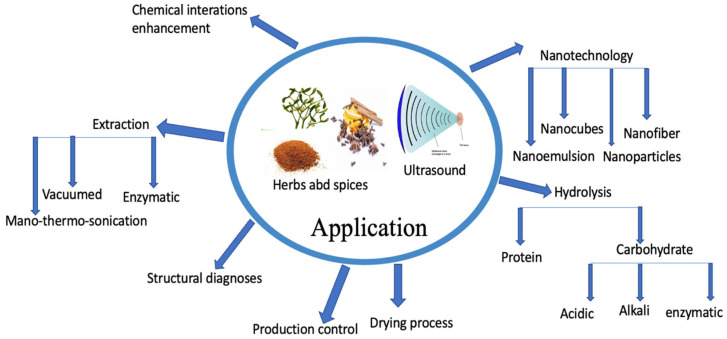
Exploring the fundamental applications linking ultrasonic technology and herbal science.

New advancements in eco-friendly ultrasound technology have been made in the field of herbal phytochemistry [108]. Figure 3 provides a summary of the typical uses of ultrasound (US) in the fields of herbal science and technology. In a separate research study conducted by Pudziuvelyte et al. [109], *Elsholtzia ciliata* herb was investigated, and it was found that the use of ultrasound technology resulted in a significant increase in the extracted apigenin phenolic yield, with a value of 855.54 μg/g compared to 141.06 μg/g using the maceration method. The authors also discovered that an 11 min US-assisted extraction process resulted in a 20% increase in the mass fraction of total phenols compared to a water bath shaker for 30 min using the same solvent. Furthermore, the US treatment method led to a chlorogenic acid content of 2174.70 μg/g after 30 min, while the percolation extraction method resulted in a lower value of 683.40 μg/g [109]. Munekata et al. [110] conducted a study to optimize ultrasound (US) parameters for extracting antioxidants from Thymus vulgaris and Rosmarinus officinalis. According to their findings, using US-assisted extraction at 400 W and 40 °C for 10 min resulted in higher extraction yields of carotenoids compared to conventional extraction using the heating under magnetic stirrer method. The authors also noted that US improved the aqueous extraction of antimicrobial compounds from thyme [110].

The ideal conditions for ultrasound (US) extraction of total flavonoids from sour jujube seeds (Caenorhabditis elegans) were determined to be 404 W and 60 °C for 60.03 min [111]. In a study, it was found that the use of US increased flavonoid yield by 17.11% compared to heat reflux extraction. Additionally, flavonoids extracted using US showed higher antioxidant capacity against DPPH, superoxide, and hydroxyl radicals due to significant differences in their chemical composition as a result of using US-assisted extraction (UAE) rather than heat reflux extraction (HRE) [111]. Table 2 illustrates how the processing of TS affects the phytochemicals and bioactive compounds in certain food matrices.

## 10. Effect of TS on Inactivation of Enzymes in Food

Enzymes are a type of protein that occur naturally and are essential for biochemical processes in living organisms, as noted by Patel et al. [118]. Nonetheless, in food, enzymes can also lead to spoilage and deterioration, which can negatively impact the quality and safety of food items, as highlighted by Dhar et al. [119]. Consequently, several techniques have been developed to neutralize or eradicate enzymes in food, as noted by Umair et al. [120]. One such approach is TS, which entails applying heat and ultrasound waves simultaneously to food, according to Rani et al. [53].

TS is a type of processing technique that does not involve heat and has become increasingly popular in the food industry due to its effectiveness in deactivating enzymes while maintaining the nutritional and sensory properties of food products, as highlighted by Urango et al. [121]. This technique involves subjecting a food sample to high-intensity ultrasound waves while heating it simultaneously, according to Chavan et al. [122]. As noted by Huang et al. [123], the application of both heat and ultrasound produces a tumultuous setting that has the potential to impair or eliminate enzymes in food, causing them to become inactive.

Numerous research studies have explored the impact of TS on enzyme deactivation in food [124]. For example, Islam et al. [124] examined how TS affected the activity of pectin methyl esterase (PME) and polygalacturonase (PG) in tomato paste. The findings revealed that TS was particularly effective in deactivating PG, with a 62% reduction in activity, while complete deactivation of PME was observed at a temperature of 70 °C and an ultrasound frequency of 20 kHz [125].

In another investigation, the efficacy of TS on Pectin methyl esterase heat-resistant fraction (PME) activity in orange juice was assessed. The findings indicated that TS was highly efficient in deactivating PME by 25 times in citrate buffer and more than 400 times in orange juice at a temperature of 72 °C and an ultrasound frequency of 20 kHz [126]. Moreover, TS has demonstrated its effectiveness in inactivating Lipase milk whey, which can cause rancidity and off-flavors in dairy products. A study found that lipase in milk was entirely inactivated by TS at a temperature range of 110–140 °C and an ultrasound frequency of 20 kHz [126,127]. 

Manas et al. [128] conducted a study using a two-step approach to inactivate lysozyme, which involved a combination of TS and mano-TS (MTS). In the first stage, ultrasonic waves were used to generate microbubbles that caused the protein to unfold in the interfacial area. Although this change in protein structure has a minor effect on enzyme activity, it makes the molecule more susceptible to denaturation by another agent, such as heat, which is used in the second stage. The process of inactivating the enzyme had two stages. Initially, the enzymatic activity decreased slowly, and subsequently, the rate of inactivation increased suddenly [128]. 

TS has several advantages over traditional thermal processing methods [129]. TS has the advantage of achieving a considerable level of enzyme inactivation while maintaining the nutritional and sensory attributes of food products. It can achieve this because it is a non-thermal processing method that requires lower temperatures than conventional thermal processing methods to inactivate enzymes [130]. Additionally, TS can reduce processing time and energy requirements, which can lead to cost savings and improved efficiency [122]. Table 3 provides an overview of the level of enzyme inactivation attained using different thermosonication conditions.

## 11. Utilizing Natural Antimicrobial Agents such as Ascorbic Acid, Nisin, and ϵ-polylysine (ϵ-PL) in Combination with TS

Hurdle technology involves combining multiple preservative factors to achieve maximum microbial lethality while minimizing any negative impact on the quality of the product. Examples of these preservative factors include heating, acidification, and the addition of preservatives, among others [138,139]. The hurdle technology was used to both kill microbes and improve the quality of soft persimmon juice during storage. The hurdle technology involved using moderate temperature, ultrasonication, and adjusting the pH with ascorbic acid. The researchers tested different combinations of these factors at various levels to determine the most effective conditions for killing Escherichia coli and Listeria monocytogenes of the soft persimmon (*Diospyros kaki T.*) juice. The best combination of hurdles was determined to be adding 1% ascorbic acid and applying TS at 50 °C for 30 min, based on both microbial inactivation and sensory [140].

Nisin is a type of antimicrobial peptide that is produced by specific strains of Lactococcus lactis subsp. lactis and is resistant to high temperatures. When consumed, it is broken down into amino acids by a protease in the digestive tract and does not cause allergic reactions or generate resistance. The World Health Organization has acknowledged it as a safe option for preserving food. The bactericidal action of nisin involves inhibiting the biosynthesis of cell walls, resulting in a sequence of events that includes ATP hydrolysis, leakage of ions, formation of pores in the membrane, disturbance of pH balance and proton motive force, and ultimately causing the death of bacterial cells [141,142]. The effects of treating orange juice with a combination of TS and nisin (TSN) or thermal processing (TP) on various aspects of quality were examined. This included investigating changes in microbial and enzyme activity, as well as evaluating any alterations in the physicochemical, nutritional, functional, and sensory properties of the juice [143]. The use of TSN treatment resulted in noticeable enhancements to the color, physicochemical properties, and sensory quality of the orange juice. Additionally, it led to a significant increase in the total polyphenols content (TPC) and total carotenoids (TC), which rose by 10.03% and 20.10%, respectively. The antioxidant capacity, as measured by ORAC and DPPH, also improved by 51.10% and 10.58%, respectively, while the levels of total flavonoids and ascorbic acid remained mostly unchanged [143].

There is a growing trend in the food industry towards the use of natural bacteriostatic agents. One such example is ϵ-polylysine (ϵ-PL), which is a polypeptide consisting of 25–35 l-lysine units. It has broad-spectrum antibacterial properties and is biodegradable, with high water solubility and thermal stability [144]. The safety of ϵ-PL has been recognized by both the USA and Japan, and it is currently used as an approved food preservative in a variety of food products, such as meat, aquatic products, starchy food, dairy products, soft drinks, and fresh-cut fruits and vegetables [144,145]. The mechanism by which ϵ-PL works involves changing the permeability of the microbial cell wall and cell membrane upon contact, leading to their destruction. Furthermore, ϵ-PL disrupts the enzyme or functional protein system of the bacteria, damages their genetic material and structure, and inhibits their respiratory systems, thereby effectively preventing bacterial growth [144,145]. The impact of TS combined with ε-polylysine (TSε) on both the microorganisms and enzyme deactivation in orange juice, as well as its overall quality, was investigated [146]. The findings indicated that TSε and HTLT treatments were effective in reducing the number of microorganisms and deactivating enzymes in orange juice. Furthermore, ε-polylysine exhibited a significant lethal effect on the total bacterial count and yeast in orange juice when used in combination with TSε treatment. Although HTLT treatment did not achieve sterilization due to the presence of *Alyciclobacillus* spores, it could be substituted with other forms of inactivation or pasteurization in cases where the *Alyciclobacillus* count is high. Additionally, TSε treatment maintained the sensory quality of the juice, which was superior to that of HTLT-treated juice. Moreover, TSε treatment significantly increased the content of functional substances in orange juice, such as polyphenols, carotenoids, and antioxidant activities, including ORAC, DPPH, and FRAP, by 12.53%, 36.46%, 53.27%, 14.42%, and 7.26%, respectively. The ascorbic acid retention rate was also high at 85.42%. Therefore, as an innovative non-thermal microbial inactivation technology, TSε treatment enhances the sensory and functional properties of orange juice [146]. The use of natural antimicrobial agents such as ascorbic acid, nisin, and ε-polylysine in combination with TS is a promising approach to enhancing the safety and shelf life of food products. Further research is needed to optimize the use of these natural antimicrobial agents and to better understand their impact on the quality and safety of food products. Natural antimicrobial agents such as ascorbic acid, nisin, and ε-polylysine can be combined with thermosonication, a food processing technique that uses heat and ultrasound to control the growth of microorganisms in food products. Ascorbic acid, nisin, and ε-polylysine work by inhibiting the growth of bacteria, and thermosonication enhances their effectiveness by increasing their solubility and penetration into food products. However, the effectiveness of these agents may be limited by their sensitivity to heat and ultrasound, and their use in food products raises concerns about their safety and potential adverse effects on human health.

There are regulatory and safety concerns associated with the use of thermosonication and natural antimicrobial agents in food products. While these techniques can be effective in reducing microbial contamination and preserving food quality, they can also have potential side effects that need to be carefully evaluated and monitored. Regulatory agencies require rigorous testing and evaluation before approving these substances for use in food, and food manufacturers must adhere to strict guidelines and regulations to ensure the safety and quality of their products. It is important to carefully evaluate the safety and effectiveness of these antimicrobial agents before using them in food processing.

To optimize the use of thermosonication and natural antimicrobial agents in food preservation, further research is needed in several areas, including identifying the optimal processing conditions, understanding the mechanisms of action, evaluating the potential development of microbial resistance, assessing the impact on sensorial and nutritional quality, and considering regulatory considerations. Such research could help address current challenges in food safety and preservation, including the demand for natural and minimally processed foods, the need to reduce chemical preservatives, and the emergence of antimicrobial-resistant microorganisms.

TS and natural antimicrobial agents can improve food preservation and safety, but their benefits must be weighed against cost, scalability, and environmental impact. To achieve this balance, a cost-benefit analysis can be conducted to determine economic feasibility, alternative sources of natural antimicrobial agents can be explored, and production processes can be optimized. Environmental impact can be mitigated by considering the entire product life cycle, using environmentally friendly materials, and recyclable packaging. Various strategies can be employed to balance these considerations.

## 12. Conclusions and Future Remark

TS is a promising technology with great potential for improving the processing, preservation, and quality of food products. This comprehensive review provided an overview of the principles and mechanisms of TS, as well as its applications in various sectors of the food industry. The review also highlighted the advantages and limitations of TS, as well as its impact on the quality and safety of food products. TS represents a novel and efficient technique for improving the quality and safety of food products, and its adoption is expected to increase in the coming years.

However, there are still some challenges and future research directions that need to be addressed to fully exploit the potential of TS. One of the major challenges is the optimization of the process parameters, including temperature, pressure, and frequency, to achieve optimal results in terms of microbial inactivation, enzyme inactivation, and preservation of the nutritional quality. Another challenge is the scalability of TS, as it requires expensive equipment and may not be suitable for large-scale production.

Additionally, combining ultrasound with natural microbicidal substances can have synergistic effects, leading to more effective and sustainable methods of food preservation. The underlying mechanisms of action, including micro-streaming and cavitation, should be emphasized to provide a detailed understanding of how these techniques can work together. Optimization and standardization are necessary to fully harness the potential of this combination in the food industry, which may involve identifying the most effective combinations of ultrasound frequency, power, and exposure time and determining the optimal concentration and composition of natural antimicrobial agents. Overall, highlighting these key phenomena can provide a more nuanced analysis of the benefits and potential applications of the combination of ultrasound with natural microbicidal substances. Overall, thermosonication shows great potential as a novel and sustainable food processing technology that can meet the increasing demand for safe and high-quality food products. Future research should focus on developing cost-effective and scalable TS systems that can be integrated into existing food processing lines.

## Figures and Tables

**Table 1:** Studies of thermosonication (TS) effects on inactivation of microorganisms in some food matrix.

Microorganism	Inactivation Conditions	Food Matrix	Findings	References
Aerobic Mesophilic (AMB) Lactic Acid Bacteria (LAB) Aerobic Psychrotrophic (APB)	20 kHz, power (160, 400, and 640 W), 72–75 °C for 15 s	Minas chees	2.22 and 2.35 log CFU/g reduction for AMB 3.30 and 3.85 log CFU/g reduction for APB 3.65 log CFU/g reduction for LAB	[89]
*Escherichia coli* (*E.coli*)	intensity 37.87 W/cm^2^, acoustic energy density 0.57 W/mL at 60 °C for 6 min	green juice	7.42 log reduction	[90]
*Bacillus subtilis*	40 kHz, 240 W, 25 min	pasteurized milk	4.09 log CFU/mL reduction	[91]
*E. coli*.K-12	(40, 50, 60) °C, 37 kHz, 150 W	pumpkin juice	6.62 ± 0.00 log cfu/mL reduction For batch TS; 6.23 ± 0.34 cfu/mL reduction for continuous TS	[92]
*Staphylococcus aureus*	frequency of 20 kHz, 80% amplitude at 50 and 60 °C ± 2 °C	orange juice	5 log cfu/mL reduction after 1 and 25 min	[93]
Total bacterial count (TBC) mold and yeast (Y&M)	(40 kHz, 400 W at 40, 50 and 60 °C each for 5, 10, 20 and 30 min)	hog plum juice	TBC reduced from 3.75 to 1.01 log CFU/mL, while Y&M reduced from 4.17 to 3.64 log CFU/mL at 40 °C. No detectable growth in the bacterial count at 50 and 60 °C, while reduced Y&M at 50 °C from 3.36 to 1.93 log CFU/mL, with no detectable growth at 60 °C.	[65]
Total aerobic mesophilic	60 °C at either 35 or 130 kHz for 5 min at	Anthocyanin-Enriched Tomato Juice	higher microbial inactivation compared to thermal pasteurisation at 80 °C for 1 min	[94]
*Alicyclobacillus acidoterrestris* spores	120–480 W power levels and 35 kHz frequency at 70, 80, 85, 90 and 95 °C for different times.	apple juice	spores decreased by 4.8, 4.7 and 5.5 log-cycles (at 85, 90 and 95 °C, respectively) after 90, 60 and 20 min.	[95]
total plate count (TPC) *E. coli*/coliforms Y&M	200 W, 400 W, and 600 W, 30 kHz, at 60 ± 1 °C for 20 min	spinach juice	Y&M, E.coli/coliform, and TPC in the TS3 sample were reduced up to 4 log CFU/mL at 600 W, 30 kHz, 60 °C for 20 min	[96]

**Table 2:** The Influence of TS Processing on Phytochemicals and Bioactive Compounds in some food matrix.

Type of Food	Treatment	Relevant Chemical Substances	Findings	References
watermelon juice	20 kHz, temperature (25–45 °C), amplitude (24.4–61.0 μm) and processing time (2–10 min)	Lycopene (LC) ascorbic acid (AA) phenolic content (TP)	The levels of AA, LC, and TP decreased significantly when the amplitude levels were increased and when the processing time reached its maximum limit.	[50]
*Citrus limon*	20 kHz, 250 W, 29 min	Phytochemical mixture	In this study, efficient nano-cubes and nanospheres of a photocatalyst were created using ultrasound with the help of C. limon LE and Ag: CdO.	[112]
almond-based beverage	three levels of acoustic power (4.6, 8.5, 14.5 W) holding time 5, 10, and 15 min,50 °C	flavonoid	The number of flavonoids present in the sample increased as a result of the TS treatments.	[113]
Apple juice	ultrasound in-bath (25 kHz, 30 min, 0.06 W cm^−3^) and ultrasound with-probe sonicator (20 kHz, 5 and 10 min, 0.30 W cm^3^) at 20, 40 and 60 °C	TP flavonoids flavonols	At a temperature of 60 °C, the ultrasound with probe method was found to be more effective than ultrasound in-bath in retaining the total phenolic, flavonoid, and flavonol content.	[114]
Blueberry juice	40 kHz and 240 W, 25 °C and 45 °C for 15 min	TP Total anthocyanins (TA)	TS at 45 °C significantly increased the TA and TP.	[115]
custard apple juice	(20 kHz, 67.84 W/cm^2^) from 0 to 40 min	TP vitamin C	Compared to the untreated juice, TS showed a rise of around 15% in TP, and the processing aid successfully maintained the vitamin C level in the juice.	[116]
grapefruit juice	(20, 30, 40, 50 and 60 °C), (28 kHz), (70%, 420 W), time (30 and 60 min)	total carotenoids (TC)	All TS treatments resulted in a significant increase compared to the control group. However, the highest increase in TC was observed in the samples processed at 60 °C for 60 min, compared to the control group.	[12]
orange-carrot juice blend	110 W, 40 kHz, 40, 50, and 60 °C, 5 and 10 min.	TP TC	All of the treatments succeeded in preserving the (TC) and TP of the juice blend.	[117]

**Table 3:** Studies of thermosonication (TS) effects on inactivation of enzymes in some food matrix.

Enzymes	Treatment	Food Matrix	Findings	References
Peroxidase	20 kHz, 50% of power, temperatures above 85 °C	Watercress	Temperatures above 85 °C and with the same blanching duration resulted in more effective enzyme deactivation compared to heat-based blanching methods.	[131]
Polyphenol oxidases Pectinmethylesterase	20 kHz, temperatures (from 44 to 67 °C), amplitude (25–100%)	Cloudy apple juice	The use of TS caused a greater deactivation of enzymes and notably improved the way particles were distributed in cloudy apple juice.	[132]
Pectin methylesterase (PME) & Polygalacturonase (PG)	20 kHz, amplitude of 65 μm and temperatures between 50 and 75 °C	Tomato juice	When temperatures were in the range of 60–75 °C, the rate at which (PME) was inactivated increased by 1.5 to 6 times, and the rate at which (PG) was inactivated increased by 2.3 to 4 times.	[133]
Polyphenol oxidase (PPO) peroxidase (POD)	Amplitude 60%, 80%, and 100% (125, 170, and 210 μm); temperatures (40, 50, and 60 °C); different times (10, 20, and 30 min).	Peach juice	Enzyme inactivation at a temperature of 60 °C, which is lower than the temperature required for complete inactivation through thermal treatment (which is 70 °C).	[134]
PME & PPO	20 kHz,1500 W, 70–85 amplitude, 40–55 °C	Fruit smoothie	The best processing conditions was found to be 77.5% amplitude, 20 min, and 47.5 C, controlling both enzymatic activities	[135]
POD	20 kHz, 50 W/cm^2^, amplitude 152 140 µm, 50–80 °C for 25 20 min	Sugarcane juice	TS was more effective than conventional treatments in enzyme inactivation at higher rates (77.3%) when temperatures of 70 and 75 °C were used.	[136]
PPO & POD	amplitude (20%, 40%, 60%, 80% and 100%) and time (1–12 min), intensity levels 90, 181, 271, 362 and 452 W/cm^2^	Bayberry juice	Inactivation rate of PPO and POD was lower when ultrasound was used with cooling compared to ultrasound treatment.	[86]
PPO	(20, 40, 60) kHz, (20, 30, 40) min, (45, 60, 75) °C, 100% of power (300 W)	Orange juice	TS was able to inactivate PPO enzymes by up to 56.17% compared to untreated juice not only due to changes in the active site of the enzyme but also to the overall structural adjustments made to the PPO enzyme.	[137]

## Data Availability

No applicable.

## References

[1] Ercan S.S., Soysal C. (2013). Use of ultrasound in food preservation. Nat. Sci..

[2] Lee H., Zhou B., Liang W., Feng H., Martin S.E. (2009). Inactivation of *Escherichia coli* cells with sonication, manosonication, thermosonication, and manothermosonication: Microbial responses and kinetics modeling. J. Food Eng..

[3] Xu J., Zhang M., Cao P., Adhikari B., Yang C. (2019). Microorganisms control and quality improvement of stewed pork with carrots using ZnO nanoparticels combined with radio frequency pasteurization. Food Biosci..

[4] Yang S., Yuan Z., Aweya J.J., Huang S., Deng S., Shi L., Zheng M., Zhang Y., Liu G. (2021). Low-intensity ultrasound enhances the antimicrobial activity of neutral peptide TGH2 against *Escherichia coli*. Ultrason. Sonochem..

[5] Kadkhodaee R., Povey M.J.W. (2008). Ultrasonic inactivation of Bacillus α-amylase. I. Effect of gas content and emitting face of probe. Ultrason. Sonochem..

[6] Gao S., Hemar Y., Ashokkumar M., Paturel S., Lewis G.D. (2014). Inactivation of bacteria and yeast using high-frequency ultrasound treatment. Water Res..

[7] Villamiel M., de Jong P. (2000). Influence of high-intensity ultrasound and heat treatment in continuous flow on fat, proteins, and native enzymes of milk. J. Agric. Food Chem..

[8] Wang S., Kang J., Zhang X., Guo Z. (2018). Dendrites fragmentation induced by oscillating cavitation bubbles in ultrasound field. Ultrasonics.

[9] Ashokkumar M. (2011). The characterization of acoustic cavitation bubbles—An overview. Ultrason. Sonochem..

[10] Costa M.G.M., Fonteles T.V., de Jesus A.L.T., Almeida F.D.L., de Miranda M.R.A., Fernandes F.A.N., Rodrigues S. (2013). High-intensity ultrasound processing of pineapple juice. Food Bioprocess Technol..

[11] Piyasena P., Mohareb E., McKellar R.C. (2003). Inactivation of microbes using ultrasound: A review. Int. J. Food Microbiol..

[12] Aadil R.M., Zeng X., Zhang Z., Wang M., Han Z., Jing H., Jabbar S. (2015). Thermosonication: A potential technique that influences the quality of grapefruit juice. Int. J. Food Sci. Technol..

[13] Alcántara-Zavala A.E., de Dios Figueroa-Cárdenas J., Pérez-Robles J.F., Arámbula-Villa G., Miranda-Castilleja D.E. (2021). Thermosonication as an alternative method for processing, extending the shelf life, and conserving the quality of pulque: A non-dairy Mexican fermented beverage. Ultrason. Sonochem..

[14] Parreiras P.M., Nogueira J.A.V., da Cunha L.R., Passos M.C., Gomes N.R., Breguez G.S., Falco T.S., Bearzoti E., Menezes C.C. (2020). Effect of thermosonication on microorganisms, the antioxidant activity and the retinol level of human milk. Food Control.

[15] Park J.J., Olawuyi I.F., Lee W.Y. (2023). Effect of combined UV-thermosonication and *Ecklonia cava* extract on advanced glycation end-products in soymilk. J. Food Process Eng..

[16] Yin H., Hao J., Zhu Y., Li Y., Wang F., Deng Y. (2019). Thermosonication and inactivation of viable putative non-culturable *Lactobacillus acetotolerans* in beer. J. Inst. Brew..

[17] Lyu C., Qi X., Ying S., Wang J. (2021). Impact of Pulsed Electric Fields Combined with Thermosonication on the Physicochemical Properties of Chinese Rice Wine. Trans. ASABE.

[18] Xu B., Feng M., Chitrakar B., Cheng J., Wei B., Wang B., Zhou C., Ma H. (2023). Multi-frequency power thermosonication treatments of clear strawberry juice: Impact on color, bioactive compounds, flavor volatiles, microbial and polyphenol oxidase inactivation. Innov. Food Sci. Emerg. Technol..

[19] Ibrahim O.O. (2020). Thermal and nonthermal food processing technologies for food preservation and their effects on food chemistry and nutritional values. EC Nutr..

[20] Hiremath L., Nipun S., Sruti O., Kala N.G., Aishwarya B.M. (2020). Sonochemistry: Applications in Biotechnology. Sonochemical Reactions.

[21] Manbachi A., Cobbold R.S.C. (2011). Development and application of piezoelectric materials for ultrasound generation and detection. Ultrasound.

[22] Liu Z., Yu X., Li L. (2020). Piezopotential augmented photo-and photoelectro-catalysis with a built-in electric field. Chin. J. Catal..

[23] Leadley C.E., Williams A. (2006). Pulsed electric field processing, power ultrasound and other emerging technologies. Food Process. Handb..

[24] Mulet A., Carcel J., Benedito J., Rosselló C., Simal S. (2016). Ultrasonic Mass Transfer Enhancement in Food Processing. Transport Phenomena in Food Processing.

[25] Mason T.J. (1998). Power Ultrasound in Food Processing—The Way Forward. Ultrasound in Food Processing.

[26] Chemat F., Khan M.K. (2011). Applications of ultrasound in food technology: Processing, preservation and extraction. Ultrason. Sonochem..

[27] Awad N.S., Paul V., AlSawaftah N.M., Ter Haar G., Allen T.M., Pitt W.G., Husseini G.A. (2021). Ultrasound-responsive nanocarriers in cancer treatment: A review. ACS Pharmacol. Transl. Sci..

[28] Chandan R., Mehta S., Banerjee R. (2020). Ultrasound-responsive carriers for therapeutic applications. ACS Biomater. Sci. Eng..

[29] Hamersky S.J. (2022). Systematic Intervention Component Analysis: Dose-Response for Therapeutic Ultrasound. Ph.D. Thesis.

[30] Huang B., Jiang Y., Zhang L., Yang B., Guo Y., Yang X., Gong P. (2023). Low-intensity pulsed ultrasound promotes proliferation and myelinating genes expression of Schwann cells through NRG1/ErbB signaling pathway. Tissue Cell.

[31] Rittmeier L., Rauter N., Mikhaylenko A., Lammering R., Sinapius M. (2023). The Guided Ultrasonic Wave Oscillation Phase Relation between the Surfaces of Plate-like Structures of Different Material Settings. Proceedings of the Acoustics.

[32] Dong Z., Delacour C., Mc Carogher K., Udepurkar A.P., Kuhn S. (2020). Continuous ultrasonic reactors: Design, mechanism and application. Materials.

[33] Liu S., Wang Y., Yang X., Lei B., Liu L., Li S.X., Ni D., Wang T. (2019). Deep learning in medical ultrasound analysis: A review. Engineering.

[34] Umar A., Atabo S. (2019). A review of imaging techniques in scientific research/clinical diagnosis. MOJ Anat. Physiol..

[35] Delaney L.J., Isguven S., Eisenbrey J.R., Hickok N.J., Forsberg F. (2022). Making waves: How ultrasound-targeted drug delivery is changing pharmaceutical approaches. Mater. Adv..

[36] Abramowicz J.S., Basseal J.M. (2020). World federation for ultrasound in medicine and biology position statement: How to perform a safe ultrasound examination and clean equipment in the context of COVID-19. Ultrasound Med. Biol..

[37] Bhargava N., Mor R.S., Kumar K., Sharanagat V.S. (2021). Advances in application of ultrasound in food processing: A review. Ultrason. Sonochem..

[38] Xiao Y., Zhang J., Fang B., Zhao X., Hao N. (2022). Acoustics-actuated microrobots. Micromachines.

[39] Deng Y., Bi H., Yin H., Yu J., Dong J., Yang M., Ma Y. (2018). Influence of ultrasound assisted thermal processing on the physicochemical and sensorial properties of beer. Ultrason. Sonochem..

[40] Silva F.V.M. (2020). Ultrasound assisted thermal inactivation of spores in foods: Pathogenic and spoilage bacteria, molds and yeasts. Trends Food Sci. Technol..

[41] Su Y., Jiang L., Chen D., Yu H., Yang F., Guo Y., Xie Y., Yao W. (2022). In vitro and in silico approaches to investigate antimicrobial and biofilm removal efficacies of combined ultrasonic and mild thermal treatment against *Pseudomonas fluorescens*. Ultrason. Sonochem..

[42] Jafarpour D. (2022). The effect of heat treatment and thermosonication on the microbial and quality properties of green olive. J. Food Meas. Charact..

[43] Nunes B.V., da Silva C.N., Bastos S.C., de Souza V.R. (2022). Microbiological inactivation by ultrasound in liquid products. Food Bioprocess Technol..

[44] Boghossian M., Brassesco M.E., Miller F.A., Silva C.L.M., Brandão T.R.S. (2023). Thermosonication Applied to Kiwi Peel: Impact on Nutritional and Microbiological Indicators. Foods.

[45] Dos Santos Rocha C., Magnani M., de Paiva Anciens Ramos G.L., Bezerril F.F., de Freitas M.Q., Cruz A.G., Pimentel T.C. (2022). Emerging technologies in food processing: Impacts on sensory characteristics and consumer perception. Curr. Opin. Food Sci..

[46] Mandha J., Shumoy H., Matemu A.O., Raes K. (2023). Characterization of fruit juices and effect of pasteurization and storage conditions on their microbial, physicochemical, and nutritional quality. Food Biosci..

[47] Tiwari B.K., O’Donnell C.P., Cullen P.J. (2009). Effect of sonication on retention of anthocyanins in blackberry juice. J. Food Eng..

[48] Valero M., Recrosio N., Saura D., Muñoz N., Martí N., Lizama V. (2007). Effects of ultrasonic treatments in orange juice processing. J. Food Eng..

[49] Cruz R.M.S., Vieira M.C., Silva C.L.M. (2008). Effect of heat and thermosonication treatments on watercress (*Nasturtium officinale*) vitamin C degradation kinetics. Innov. Food Sci. Emerg. Technol..

[50] Rawson A., Tiwari B.K., Patras A., Brunton N., Brennan C., Cullen P.J., O’Donnell C. (2011). Effect of thermosonication on bioactive compounds in watermelon juice. Food Res. Int..

[51] Wu J., Gamage T.V., Vilkhu K.S., Simons L.K., Mawson R. (2008). Effect of thermosonication on quality improvement of tomato juice. Innov. Food Sci. Emerg. Technol..

[52] Urango A.C.M., Strieder M.M., Silva E.K., Meireles M.A.A. (2021). Thermosonication process design for recovering bioactive compounds from fennel: A comparative study with conventional extraction techniques. Appl. Sci..

[53] Rani M., Sood M., Bandral J.D., Bhat A., Gupta I. (2020). Thermosonication technology and its application in food industry. Int. J. Chem. Stud..

[54] Aadil R.M., Khalil A.A., Rehman A., Khalid A., Inam-ur-Raheem M., Karim A., Gill A.A., Abid M., Afraz M.T. (2020). Assessing the impact of ultra-sonication and thermo-ultrasound on antioxidant indices and polyphenolic profile of apple-grape juice blend. J. Food Process. Preserv..

[55] Ye L., Zhu X., Liu Y. (2019). Numerical study on dual-frequency ultrasonic enhancing cavitation effect based on bubble dynamic evolution. Ultrason. Sonochem..

[56] Pandey T., Sandhu A., Sharma A., Ansari M.J. (2023). Recent advances in applications of sonication and microwave. Ultrasound Microw. Food Process..

[57] Shirsath S.R., Sonawane S.H., Gogate P.R. (2012). Intensification of extraction of natural products using ultrasonic irradiations—A review of current status. Chem. Eng. Process. Process Intensif..

[58] Cano-Lamadrid M., Artés-Hernández F. (2022). Thermal and Non-Thermal Treatments to Preserve and Encourage Bioactive Compounds in Fruit-and Vegetable-Based Products. Foods.

[59] Naik M., Natarajan V., Thangaraju S., Modupalli N., Rawson A. (2022). Assessment of storage stability and quality characteristics of thermo-sonication assisted blended bitter gourd seed oil and sunflower oil. J. Food Process. Eng..

[60] Chemat F., Rombaut N., Sicaire A.-G., Meullemiestre A., Fabiano-Tixier A.-S., Abert-Vian M. (2017). Ultrasound assisted extraction of food and natural products. Mechanisms, techniques, combinations, protocols and applications. A review. Ultrason. Sonochem..

[61] Yusaf T., Al-Juboori R.A. (2014). Alternative methods of microorganism disruption for agricultural applications. Appl. Energy.

[62] Bermúdez-Aguirre D., Mobbs T., Barbosa-Cánovas G.V. (2011). Ultrasound applications in food processing. Ultrasound Technol. Food Bioprocess..

[63] Ravikumar M., Suthar H., Desai C., Gowda S.A. (2017). Ultrasonication: An advanced technology for food preservation. Int. J. Pure Appl. Biosci..

[64] Basumatary B., Nayak P.K., Chandrasekar C.M., Nath A., Nayak M., Kesavan R.K. (2020). Impact of thermo sonication and pasteurization on the physicochemical, microbiological and anti-oxidant properties of pomelo (*Citrus maxima*) juice. Int. J. Fruit Sci..

[65] Oladunjoye A.O., Adeboyejo F.O., Okekunbi T.A., Aderibigbe O.R. (2021). Effect of thermosonication on quality attributes of hog plum (*Spondias mombin* L.) juice. Ultrason. Sonochem..

[66] Kesavan R.K., Gogoi S., Nayak P.K. (2023). Influence of thermosonication and pasteurization on the quality attributes of kutkura (*Meyna spinosa*) juice. Appl. Food Res..

[67] Gursoy O., Yilmaz Y., Gokce O., Ertan K. (2016). Effect of ultrasound power on physicochemical and rheological properties of yoghurt drink produced with thermosonicated milk. Emir. J. Food Agric..

[68] Inguglia E.S., Granato D., Kerry J.P., Tiwari B.K., Burgess C.M. (2020). Ultrasound for meat processing: Effects of salt reduction and storage on meat quality parameters. Appl. Sci..

[69] Peña-Gonzalez E., Alarcon-Rojo A.D., Garcia-Galicia I., Carrillo-Lopez L., Huerta-Jimenez M. (2019). Ultrasound as a potential process to tenderize beef: Sensory and technological parameters. Ultrason. Sonochem..

[70] Bermudez-Aguirre D., Niemira B.A. (2022). Pasteurization of Foods with Ultrasound: The Present and the Future. Appl. Sci..

[71] Desphande V. (2020). Effect of Thermosonication on Viscosity of Milk Concentrates and Milk Quality and Shelf Life. Ph.D. Thesis.

[72] Kutlu N., Pandiselvam R., Kamiloglu A., Saka I., Sruthi N.U., Kothakota A., Socol C.T., Maerescu C.M. (2022). Impact of ultrasonication applications on color profile of foods. Ultrason. Sonochem..

[73] Nowacka M., Dadan M., Janowicz M., Wiktor A., Witrowa-Rajchert D., Mandal R., Pratap-Singh A., Janiszewska-Turak E. (2021). Effect of nonthermal treatments on selected natural food pigments and color changes in plant material. Compr. Rev. Food Sci. Food Saf..

[74] Raso J., Álvarez Lanzarote I. (2018). Aplicación de Ultrasonidos en el Cocinado de Alimentos. Ph.D. Thesis.

[75] Chitgar M.F., Aalami M., Milani E., Maghsoudlo Y. (2017). Effect of TS on quality properties of barberry (*Berberis vulgaris*) juice. Iran. Food Sci. Technol. Res. J..

[76] Santhirasegaram V., Razali Z., Somasundram C. (2013). Effects of thermal treatment and sonication on quality attributes of Chokanan mango (*Mangifera indica* L.) juice. Ultrason. Sonochem..

[77] Lee H.S., Coates G.A. (2003). Effect of thermal pasteurization on Valencia orange juice color and pigments. LWT-Food Sci. Technol..

[78] Tiwari B.K., Muthukumarappan K., O’donnell C.P., Cullen P.J. (2008). Colour degradation and quality parameters of sonicated orange juice using response surface methodology. LWT Food Sci. Technol..

[79] Chen B.H., Peng H.Y., Chen H.E. (1995). Changes of carotenoids, color, and vitamin A contents during processing of carrot juice. J. Agric. Food Chem..

[80] Ibarz A., Pagán J., Panadés R., Garza S. (2005). Photochemical destruction of color compounds in fruit juices. J. Food Eng..

[81] Menis-Henrique M.E.C. (2020). Methodologies to advance the understanding of flavor chemistry. Curr. Opin. Food Sci..

[82] Salehi F. (2020). Physico-chemical properties of fruit and vegetable juices as affected by ultrasound: A review. Int. J. Food Prop..

[83] Bui A.T.H., Cozzolino D., Zisu B., Chandrapala J. (2020). Effects of high and low frequency ultrasound on the production of volatile compounds in milk and milk products—A review. J. Dairy Res..

[84] Hashemi S.M.B., Jafarpour D., Soto E.R., Barba F.J. (2023). Ultrasound-Assisted Lactic Acid Fermentation of Bakraei (*Citrus reticulata* cv. Bakraei) Juice: Physicochemical and Bioactive Properties. Fermentation.

[85] Ragab E.S., Zhang S., Korma S.A., Buniowska-Olejnik M., Nasser S.A.A., Esatbeyoglu T., Lv J., Nassar K.S. (2023). Physicochemical and Rheological Properties of Stirred Yoghurt during Storage Induced from High-Intensity Thermosonicated Goat and Cow Milk. Fermentation.

[86] Cao X., Cai C., Wang Y., Zheng X. (2018). The inactivation kinetics of polyphenol oxidase and peroxidase in bayberry juice during thermal and ultrasound treatments. Innov. Food Sci. Emerg. Technol..

[87] Guimarães J.T., Scudino H., Ramos G.L.P.A., Oliveira G.A.R., Margalho L.P., Costa L.E.O., Freitas M.Q., Duarte M.C.K.H., Sant’Ana A.S., Cruz A.G. (2021). Current applications of high-intensity ultrasound with microbial inactivation or stimulation purposes in dairy products. Curr. Opin. Food Sci..

[88] Meroni D., Djellabi R., Ashokkumar M., Bianchi C.L., Boffito D.C. (2021). Sonoprocessing: From concepts to large-scale reactors. Chem. Rev..

[89] Scudino H., Guimarães J.T., Moura R.S., Ramos G.L.P.A., Pimentel T.C., Cavalcanti R.N., Sobral L.A., Silva M.C., Mársico E.T., Esmerino E.A. (2023). Thermosonication as a pretreatment of raw milk for Minas frescal cheese production. Ultrason. Sonochem..

[90] Oner M.E. (2020). The effect of high-pressure processing or thermosonication in combination with nisin on microbial inactivation and quality of green juice. J. Food Process. Preserv..

[91] Yang S., Piao Y., Li X., Mu D., Ji S., Wu R., Wu J. (2023). A new decontamination method for Bacillus subtilisin pasteurized milk: Thermosonication treatment. Food Res. Int..

[92] Demir H., Kılınç A. (2019). Effect of batch and continuous thermosonication on the microbial and physicochemical quality of pumpkin juice. J. Food Sci. Technol..

[93] Tahi A.A., Sousa S., Madani K., Silva C.L.M., Miller F.A. (2021). Ultrasound and heat treatment effects on *Staphylococcus aureus* cell viability in orange juice. Ultrason. Sonochem..

[94] Lafarga T., Ruiz-Aguirre I., Abadias M., Viñas I., Bobo G., Aguiló-Aguayo I. (2019). Effect of thermosonication on the bioaccessibility of antioxidant compounds and the microbiological, physicochemical, and nutritional quality of an anthocyanin-enriched tomato juice. Food Bioprocess Technol..

[95] Tremarin A., Canbaz E.A., Brandão T.R.S., Silva C.L.M. (2019). Modelling *Alicyclobacillus acidoterrestris* inactivation in apple juice using thermosonication treatments. LWT.

[96] Manzoor M.F., Xu B., Khan S., Shukat R., Ahmad N., Imran M., Rehman A., Karrar E., Aadil R.M., Korma S.A. (2021). Impact of high-intensity thermosonication treatment on spinach juice: Bioactive compounds, rheological, microbial, and enzymatic activities. Ultrason. Sonochem..

[97] Barbosa-Cánovas G.V., Donsi F., Yildiz S., Candoğan K., Pokhrel P.R., Guadarrama-Lezama A.Y. (2022). Nonthermal processing technologies for stabilization and enhancement of bioactive compounds in foods. Food Eng. Rev..

[98] Wani M.Y., Ganie N.A., Wani D.M., Wani A.W., Dar S.Q., Khan A.H., Khan N.A., Manzar M.S., Dehghani M.H. (2023). The phenolic components extracted from mulberry fruits as bioactive compounds against cancer: A review. Phyther. Res..

[99] Fu Y., Liu W., Soladoye O.P. (2021). Towards innovative food processing of flavonoid compounds: Insights into stability and bioactivity. LWT.

[100] Sanjaya Y.A., Tola P.S., Rahmawati R. (2022). Ultrasound-assisted Extraction as a Potential Method to Enhanced Extraction of Bioactive Compound. Nusant. Sci. Technol. Proc..

[101] Starek A., Kobus Z., Sagan A., Chudzik B., Pawłat J., Kwiatkowski M., Terebun P., Andrejko D. (2021). Influence of ultrasound on selected microorganisms, chemical and structural changes in fresh tomato juice. Sci. Rep..

[102] Gavahian M., Manyatsi T.S., Morata A., Tiwari B.K. (2022). Ultrasound-assisted production of alcoholic beverages: From fermentation and sterilization to extraction and aging. Compr. Rev. Food Sci. Food Saf..

[103] Priyadarshini A., Rayaguru K., Nayak P.K. (2022). Effect of Ohmic Heating and TS on the Physio-Chemical, Antioxidant, Microbial and Sensory Properties of Mango Juice. Indian J. Nutr. Diet..

[104] Yildiz G., Feng H. (2019). Sonication of cherry juice: Comparison of different sonication times on color, antioxidant activity, total phenolic and ascorbic acid content. Lat. Am. Appl. Res. Int. J..

[105] Xia T., Shi S., Wan X. (2006). Impact of ultrasonic-assisted extraction on the chemical and sensory quality of tea infusion. J. Food Eng..

[106] Qin Y., Yuan Z., Yang F., Yu Y. (2022). Development of a new type of Anhua black tea and its application: Black tea wine. J. Food Process. Preserv..

[107] Raghunath S., Mallikarjunan K. (2020). Optimization of ultrasound-assisted extraction of cold-brewed black tea using response surface methodology. J. Food Process Eng..

[108] Gouda M., Bekhit A.E.-D., Tang Y., Huang Y., Huang L., He Y., Li X. (2021). Recent innovations of ultrasound green technology in herbal phytochemistry: A review. Ultrason. Sonochem..

[109] Pudziuvelyte L., Jakštas V., Ivanauskas L., Laukevičienė A., Ibe C.F.D., Kursvietiene L., Bernatoniene J. (2018). Different extraction methods for phenolic and volatile compounds recovery from Elsholtzia ciliata fresh and dried herbal materials. Ind. Crops Prod..

[110] Munekata P.E.S., Alcántara C., Žugčić T., Abdelkebir R., Collado M.C., García-Pérez J.V., Jambrak A.R., Gavahian M., Barba F.J., Lorenzo J.M. (2020). Impact of ultrasound-assisted extraction and solvent composition on bioactive compounds and in vitro biological activities of thyme and rosemary. Food Res. Int..

[111] Yang T., Fang L., Lin T., Li J., Zhang Y., Zhou A., Xie J. (2019). Ultrasonicated sour Jujube seed flavonoids extract exerts ameliorative antioxidant capacity and reduces Aβ-induced toxicity in *Caenorhabditis elegans*. J. Ethnopharmacol..

[112] Jha M., Ansari S., Shimpi N.G. (2019). Ultrasonic assisted green synthesis of Ag: CdO nanocubes and nanospheres using *Citrus limon* leaves for efficient degradation of organic dyes. J. Ind. Eng. Chem..

[113] Strieder M.M., Neves M.I.L., Belinato J.R., Silva E.K., Meireles M.A.A. (2022). Impact of thermosonication processing on the phytochemicals, fatty acid composition and volatile organic compounds of almond-based beverage. LWT.

[114] De Pinho Ferreira Guiné R., Barroca M.J. (2014). Influence of processing and storage on fruit juices phenolic compounds. Int. J. Med. Biol. Front..

[115] Chen T., Li B., Shu C., Tian J., Zhang Y., Gao N., Cheng Z., Xie X., Wang J. (2022). Combined effect of thermosonication and high hydrostatic pressure on bioactive compounds, microbial load, and enzyme activities of blueberry juice. Food Sci. Technol. Int..

[116] Dabir M.P., Ananthanarayan L. (2017). Effect of thermosonication on peroxidase, pectin methylesterase activities and on bioactive compounds in custard apple juice. J. Food Meas. Charact..

[117] Lepaus B.M., de Oliveira Santos A.K.P., Spaviero A.F., Daud P.S., de São José J.F.B. (2023). Thermosonication of Orange-Carrot Juice Blend: Overall Quality during Refrigerated Storage, and Sensory Acceptance. Molecules.

[118] Patel A.K., Dong C.-D., Chen C.-W., Pandey A., Singhania R.R. (2023). Production, Purification, and Application of Microbial Enzymes. Biotechnology of Microbial Enzymes.

[119] Dhar R., Basak S., Chakraborty S. (2022). Pasteurization of fruit juices by pulsed light treatment: A review on the microbial safety, enzymatic stability, and kinetic approach to process design. Compr. Rev. Food Sci. Food Saf..

[120] Umair M., Jabeen S., Ke Z., Jabbar S., Javed F., Abid M., Khan K.R., Ji Y., Korma S.A., El-Saadony M.T. (2022). Thermal treatment alternatives for enzymes inactivation in fruit juices: Recent breakthroughs and advancements. Ultrason. Sonochem..

[121] Urango A.C.M., Strieder M.M., Silva E.K., Meireles M.A.A. (2022). Impact of thermosonication processing on food quality and safety: A review. Food Bioprocess Technol..

[122] Chavan P., Sharma P., Sharma S.R., Mittal T.C., Jaiswal A.K. (2022). Application of high-intensity ultrasound to improve food processing efficiency: A review. Foods.

[123] Huang G., Chen S., Dai C., Sun L., Sun W., Tang Y., Xiong F., He R., Ma H. (2017). Effects of ultrasound on microbial growth and enzyme activity. Ultrason. Sonochem..

[124] Islam M.N., Zhang M., Adhikari B. (2014). The inactivation of enzymes by ultrasound—A review of potential mechanisms. Food Rev. Int..

[125] Vercet A., Sánchez C., Burgos J., Montañés L., Buesa P.L. (2002). The effects of manothermosonication on tomato pectic enzymes and tomato paste rheological properties. J. Food Eng..

[126] Vercet A., Lopez P., Burgos J. (1999). Inactivation of heat-resistant pectinmethylesterase from orange by manothermosonication. J. Agric. Food Chem..

[127] Mukhtar K., Nabi B.G., Arshad R.N., Roobab U., Yaseen B., Ranjha M.M., Aadil R.M., Ibrahim S.A. (2022). Potential Impact of Ultrasound, Pulsed Electric Field, High-Pressure Processing, Microfludization Against Thermal Treatments Preservation Regarding Sugarcane Juice (*Saccharum officinarum*). Ultrason. Sonochem..

[128] Manas P., Munoz B., Sanz D., Condon S. (2006). Inactivation of lysozyme by ultrasonic waves under pressure at different temperatures. Enzym. Microb. Technol..

[129] Ramteke S.P., Desale R.J., Kankhare D.H., Fulpagare Y.G. (2020). Thermosonication technology in the dairy industry: A review. Int. J. Adv. Res. Biol. Sci..

[130] Kar S., Sutar P.P. (2022). Enhancing the efficacy of microwave blanching-cum-black mould inactivation of whole garlic (*Allium sativum* L.) bulbs using ultrasound: Higher inactivation of peroxidase, polyphenol oxidase, and aspergillus niger at lower processing temperatures. Food Bioprocess Technol..

[131] Cruz R.M.S., Vieira M.C., Silva C.L.M. (2006). Effect of heat and thermosonication treatments on peroxidase inactivation kinetics in watercress (*Nasturtium officinale*). J. Food Eng..

[132] Illera A.E., Sanz M.T., Benito-Román O., Varona S., Beltrán S., Melgosa R., Solaesa A.G. (2018). Effect of thermosonication batch treatment on enzyme inactivation kinetics and other quality parameters of cloudy apple juice. Innov. Food Sci. Emerg. Technol..

[133] Terefe N.S., Gamage M., Vilkhu K., Simons L., Mawson R., Versteeg C. (2009). The kinetics of inactivation of pectin methylesterase and polygalacturonase in tomato juice by thermosonication. Food Chem..

[134] Baltacıoğlu H. (2022). Thermosonication of peach juice: Investigation of PPO and POD activities, physicochemical and bioactive compounds changes, and development of FT-IR–based chemometric models for the evaluation of quality. Int. J. Food Sci. Technol..

[135] Amador-Espejo G.G., Chávez-Ocegueda J., Cruz-Cansino N., Suárez-Jacobo A., Gutiérrez-Martínez P., Valencia-Flores D., Velázquez Estrada R. (2020). Thermosonication parameter effects on physicochemical changes, microbial and enzymatic inactivation of fruit smoothie. J. Food Sci. Technol..

[136] De Medeiros J.K., Sarkis J.R., Jaeschke D.P., Mercali G.D. (2021). Thermosonication for peroxidase inactivation in sugarcane juice. LWT.

[137] Wahia H., Zhou C., Sarpong F., Mustapha A.T., Liu S., Yu X., Li C. (2019). Simultaneous optimization of *Alicyclobacillus acidoterrestris* reduction, pectin methylesterase inactivation, and bioactive compounds enhancement affected by thermosonication in orange juice. J. Food Process. Preserv..

[138] Singh S., Shalini R. (2016). Effect of hurdle technology in food preservation: A review. Crit. Rev. Food Sci. Nutr..

[139] Gastélum G.G., Avila-Sosa R., López-Malo A., Palou E. (2012). Listeria innocua multi-target inactivation by TS and vanillin. Food Bioprocess Technol..

[140] Park J.J., Olawuyi I.F., Lee W.Y. (2021). Influence of TS and ascorbic acid treatment on microbial inactivation and shelf-life extension of soft persimmon (*Diospyros kaki* T.) juice. Food Bioprocess Technol..

[141] Ma T., Wang J., Wang L., Yang Y., Yang W., Wang H., Lan T., Zhang Q., Sun X. (2020). Ultrasound-combined sterilization technology: An effective sterilization technique ensuring the microbial safety of grape juice and significantly improving its quality. Foods.

[142] Mok J.H., Pyatkovskyy T., Yousef A., Sastry S.K. (2020). Synergistic effects of shear stress, moderate electric field, and nisin for the inactivation of *Escherichia coli* K12 and *Listeria innocua* in clear apple juice. Food Control.

[143] Zhao Q., Yuan Q., Gao C., Wang X., Zhu B., Wang J., Sun X., Ma T. (2021). Thermosonication combined with natural antimicrobial nisin: A potential technique ensuring microbiological safety and improving the quality parameters of orange juice. Foods.

[144] Lin L., Xue L., Duraiarasan S., Haiying C. (2018). Preparation of ε-polylysine/chitosan nanofibers for food packaging against Salmonella on chicken. Food Packag. Shelf Life.

[145] Na S., Kim J.-H., Jang H.-J., Park H.J., Oh S.-W. (2018). Shelf life extension of Pacific white shrimp (*Litopenaeus vannamei*) using chitosan and ε-polylysine during cold storage. Int. J. Biol. Macromol..

[146] Sun X., Zhao Q., Yuan Q., Gao C., Ge Q., Li C., Liu X., Ma T. (2022). Thermosonication combined with ε-polylysine (TSε): A novel technology to control the microbial population and significantly improve the overall quality attributes of orange juice. Food Control.

